# Extending the electric vehicle battery first life: Performance beyond the current end of life threshold

**DOI:** 10.1016/j.heliyon.2024.e26066

**Published:** 2024-02-13

**Authors:** Maite Etxandi-Santolaya, Lluc Canals Casals, Cristina Corchero

**Affiliations:** a Catalonia Institute for Energy Research (IREC), Energy Systems Analytics Group, Jardins de les Dones de Negre 1, 2, 08930 Sant Adrià de Besòs, Barcelona, Spain; b Department of Engineering Projects and Construction, Universitat Politècnica de Catalunya-UPC, Jordi Girona 31, 08034, Barcelona, Spain; c Bamboo Energy, Roc Boronat 117, 08018, Barcelona, Spain

**Keywords:** Electric vehicle, State of health, End of life, Internal resistance

## Abstract

Presently, Electric Vehicle batteries are considered to have reached the End of Life once their State of Health falls to 70-80%. However, this criteria is universal to all battery capacities and not based on the specific application requirements. To evaluate whether the End of Life can be extended below the current threshold, the impact of the Internal Resistance increase needs to be addressed. In this sense, this study employs a degradation aware electrothermal model to evaluate the battery performance for different use cases. The findings reveal that capacity constraints are the main cause of the End of Life, followed by power constraints. However, this is highly dependent on the battery capacity. Large capacity batteries tend to reach the End of Life for capacity constraints, whereas smaller ones show power limitations first. The temperature increase has not shown to be a restriction for any of the cases simulated. The decline in performance is for most cases (37.5% of the simulated ones) noticed below 70% State of Health, supporting that the first-life of most batteries can be extended without compromising the vehicle's performance. This is especially the case for most average drivers using large battery capacities, currently emerging on the market. The methodology proposed for the simulated cases can be extended to real time operation in the Battery Management System. Estimating the End of Life in this way can support the maximization of the first-life and only requires an appropriate use of the available data.

## 1 Introduction

The Electric Vehicle (EV) has rapidly gained popularity in recent years as a cleaner and more sustainable mode of transportation. With the world's increasing concern over climate change, governments and automakers alike have made efforts to reduce carbon emissions. Investment efforts and policies indicate that the EV is becoming the medium-term bet for the future transportation sector. As a result, the number of manufactured EVs is growing exponentially [1].

Batteries play a critical role in EVs, as they account for a significant proportion of both the vehicle's cost [2] and the environmental impact of its production [3]. Currently, most EVs use Lithium-ion (Li-ion) batteries, which have high energy density and long cycle life, making them well-suited for EVs [4]. However, the materials required to manufacture these batteries, such as lithium, cobalt and nickel, are scarce and can be environmentally damaging to extract [5]. As the electrification of the transportation sector advances, the demand for these materials will increase, making it crucial to develop sustainable and ethical supply chains for the materials, within a circular economy. Implementing a circular economy for EV batteries is essential for making EVs more sustainable and reducing their environmental impact [6].

Several initiatives are already underway to promote circularity in the battery industry. For example, second-life applications are being encouraged as a way to extend the usage of the battery instead of disposing it [7]. Additionally, recycling programs are being developed to recover materials from used batteries, reducing the need for material extraction [8]. However, before reusing and recycling, one of the primary principles of the circular economy is to promote prolonging the use of the product for as long as possible [9]. For EVs, this takes the form of extending the End of Life (EoL) to avoid early retirement [10].

There are several reasons why an EV battery reaches the EoL. In some cases, battery retirement is inevitable due to an accident or when the EV itself has reached its EoL. In other cases, the battery may no longer be safe or functional for its intended application due to degradation that negatively impacts its performance. In such cases, the extent of battery degradation should determine whether it is safe to use and effective for its intended purpose. However, the state of the art on EoL estimation does not follow this approach and assumes a universal threshold regardless of the application requirements and nominal battery capacity. The simplistic nature of this assumption is found in different areas and hinders the deployment of sustainable battery management strategies, as will be reviewed in the next section.

### 1.1 Literature review

The current criteria used to estimate the EoL of EV batteries assumes that they are no longer suitable for the automotive use once their State of Health (SoH) reaches 70-80%, which following the standard definition means that the capacity has faded between a 30-20%. This criterion, established during a period when battery technologies were markedly distinct from today [11], should be put under review in light of advancing EV technology. The fact that EV battery warranties have been extended over time, currently at 10 years for most cases, serves as evidence of the industry's increasing trust in their durability. In 2030 it is expected that the average EV lifespan will be 15 years [12], which will impact the SoH of batteries at retirement.

The 70-80% EoL threshold is deeply rooted in the literature and influences various methodologies and studies (see Table 1). Algorithms for Remaining Useful Life (RUL) estimation, critical for maintenance, oversimplify the task by assuming that, regardless of the battery and specific driving requirements, all batteries will reach the EoL at 70-80%. This threshold, which often focus solely on capacity loss, neglects crucial factors such as user-specific driving requirements, environmental conditions, and the multifaceted nature of battery underperformance. As can be seen in Table 1, only a single study proposed an application dependent EoL threshold based on driving requirements [13].

**Table 1:** Reviewed studies that are sensitive to the EoL criteria considered.

Ref	Authors	Year	Study Type	EoL criteria for 1st life
[18]	Nuhic et al.	2013	RUL	80% SoH
[19]	Dong et al.	2014	RUL	70-75% SoH
[20]	Qu et al.	2019	RUL	70% SoH
[21]	Lin et al.	2020	RUL	80% SoH
[22]	Gou et al.	2020	RUL	80% SoH
[23]	Yang et al.	2021	RUL	80% SoH
[24]	Catelani et al.	2021	RUL	70% SoH
[13]	Arrinda et al	2021	RUL	Application-dependent
[25]	Bamati and Chaoui	2022	RUL	70% SoH
[26]	Khaleghi et al.	2022	RUL	80% SoH
[27]	Assunçao et al.	2016	Second-life assessment	70% SoH
[28]	Saez-de-Ibarra et al.	2016	Second-life assessment	80% SoH
[29]	Madlener and Kirmas	2017	Second-life assessment	80% SoH
[30]	Ioakimidis et al.	2019	Second-life assessment	80% SoH
[15]	Wu et al.	2020	Second-life assessment	90-65% SoH
[31]	Haram et al.	2021	Second-life assessment	80% SoH
[32]	Fallah and Fitzpatrick	2022	Second-life assessment	90-60% SoH
[33]	Song et al.	2022	Second-life assessment	80% SoH
[34]	Salek et al.	2022	Second-life assessment	80% SoH
[35]	Terkes et al.	2023	Second-life assessment	70-80% SoH
[36]	Ai et al.	2019	Battery stock projections	Different lifespans
[37]	Jiang et al.	2021	Battery stock projections	Different lifespans but 70% SoH
[14]	Fallah et al.	2021	Battery stock projections	80% SoH
[38]	Sanclemente Crespo et al.	2022	Battery stock projections	Different lifespans but 80% SoH
[17]	Canals et al.	2022	Battery stock projections	Based on expected EV lifespan

Economic and environmental assessments exploring the potential second-life applications of batteries invariably rely on the assumption that batteries will retain 70-80% of their original capacity. Any deviations from this value will have an impact on the expected lifespan during the second-life and therefore, in the cost feasibility of the application. Out of the reviewed works (Table 1) some considered a broader range of EoL conditions (i.e. 60-90% [14] or 65-90% [15]). However, this range is assumed and not linked to any study or dependent on the battery capacity. In a recent study, actual reused batteries were tested showing an upper limit of 70% SoH [16]. It was concluded that the expected health of the batteries would be below that threshold in many cases. The study was conducted using old Nissan Leaf batteries, which have battery capacities well below the current ones.

Even broader projections concerning the future stock of EoL batteries, crucial for waste management and resource planning, rest upon this standardized value. Many of these studies assume an extended lifespan for EVs due to technological advancements but paradoxically maintain the established EoL threshold. In this regard, one study showed the difference in the expected EoL SoH based on the nominal capacity, arguing that large capacity batteries are expected to reach the EoL at healthier states than reflected by the fixed threshold [17].

In the pursuit of sustainable decision-making processes, an improved understanding of EoL SoH is imperative. Accurate estimations demand a departure from generalized assumptions and require an individualized analysis of driving requirements and conditions. This way it is possible to understand the practical limit where the battery will underperform for a practical user and prioritize actions that can maximize the battery usage and decrease its environmental impact, as will be reflected in the discussion of this study.

### 1.2 Work overview, research gap and key novelty

In this context, this work aims at increasing the understanding of practical EoL constraints for EV batteries.

The first step is to consider the capacity requirements during driving. Each EV driver requires a different amount of battery capacity to cover their driving trips depending on factors like the driving distances, charging behaviour and specific energy consumption, which is affected by the ambient temperature and road type among other aspects [39]. For example, a driver with a short daily commute may not require a large EoL capacity, while a driver who frequently takes long trips or has limited access to charging infrastructure may require a larger one. Moreover, the current EoL threshold is universal for all nominal battery capacities that can range from 16 kWh to over 100 kWh. For the same application, a loss of 20% of capacity may not pose a problem for a large-capacity battery but might force the EoL for a low-capacity one. Previous studies analysed the capacity requirements for different use cases with diverse driving times, road types, ambient temperature conditions and nominal battery capacities suggesting that, for most cases, that the energy needed to meet common trips is well beyond the battery capacity of common EVs [40]. This goes in line with other research that has examined the capacity needed to meet common trips from real data [41].

Nevertheless, another important limitation remains to be addressed to be able to define an EoL that maximizes the battery use. The capacity fade caused by battery degradation comes along an increase in the battery Internal Resistance (IR), that also affects the safety and functionality of the battery and should be considered in the EoL estimation.

The IR of a battery can rise for various reasons, including battery ageing, temperature, overcharging, and overdischarging [42]. As a battery ages, a thin film called the Solid Electrolyte Interface (SEI) layer grows on the electrode surfaces due to chemical reactions between the electrolyte and the electrode [43]. This layer can act as an additional resistance in the battery, ultimately increasing the IR. An increase in the IR can have various implications for the battery performance. On the one hand, it directly reduces the power that the battery can charge or discharge without exceeding its operating limits, as the voltage drop caused by the flow of current in the battery is proportional to the IR. This reduction can affect the acceleration, ability to drive uphill, regenerative braking capabilities and charging times. Furthermore, higher IR values increase the heat generation rate [44], which can lead to safety issues and decrease the overall efficiency of the battery due to the additional cooling required.

Understanding the impact of the increased IR in meeting the driving power requirements is key to define the EoL adequately. To the authors' knowledge a single study has addressed the underperformance derived from a limited power in the battery [45]. The study, however, was limited to a single battery capacity (24 kWh), which is far from current models on the market, and reflected driving habits of the population of the United States. In addition, due to the recent emergence of high-range EVs, very few of these vehicles have reached their EoL, resulting in a scarcity of data and information about their long-term performance. Therefore, further analysis is still required to support a data-driven EoL definition that maximizes the battery lifetime. The aim of this study is indeed to cover the existing research gap by presenting the functional EoL for a variety of use cases. This allows to showcase the way the EoL should be redefined and prove that, in a large number of cases, the first-life can be extended only with the use of the available EV data.

Due to manufacturer confidentiality, accessing EV data can be challenging. This difficulty encourages the use of simulations that can represent realistic driving conditions and be used to analyse the EoL constraints for a wide variety of scenarios. In this study, different use cases representing a variety of driving requirements, nominal battery capacities and two environmental conditions are simulated based on known electrical and thermal battery models. As discussed in the study, the methodology followed can be translated to the real-life by replacing the simulation results purely with the data coming from the Battery Management System (BMS). This highlights how a data-driven approach applied to each individual EV can be extremely beneficial in extending the battery lifetime and reducing its environmental impact.

Therefore, the novelty of the work is the analysis of performance limitations derived from the battery degradation and the calculation of the SoH value where either capacity, power or safety constraints appear for each case, which allows to define the functional EoL individually. The major contributions of the paper are listed below:

• Evaluation of the impact of the road type, climate, average driving times and nominal battery capacity in the functionality of the battery.
• Detection of the degradation level where power limitations appear.
• Identification of the constraint (capacity, power or safety) that forces the EoL.
• Evaluation of the adequacy of the commonly assumed fixed EoL threshold for representative use cases.
• Proposed methodology to estimate the EoL in real-life based on onboard data.

The paper is divided as follows. The methodology is presented in Section 2, where the electrical and thermal models and the use case definition is included. Section 3 provides the simulations results and Section 4 the discussion of the results. The main conclusions of the paper are extracted in Section 5.

## 2 Methodology

**Figure 1:**
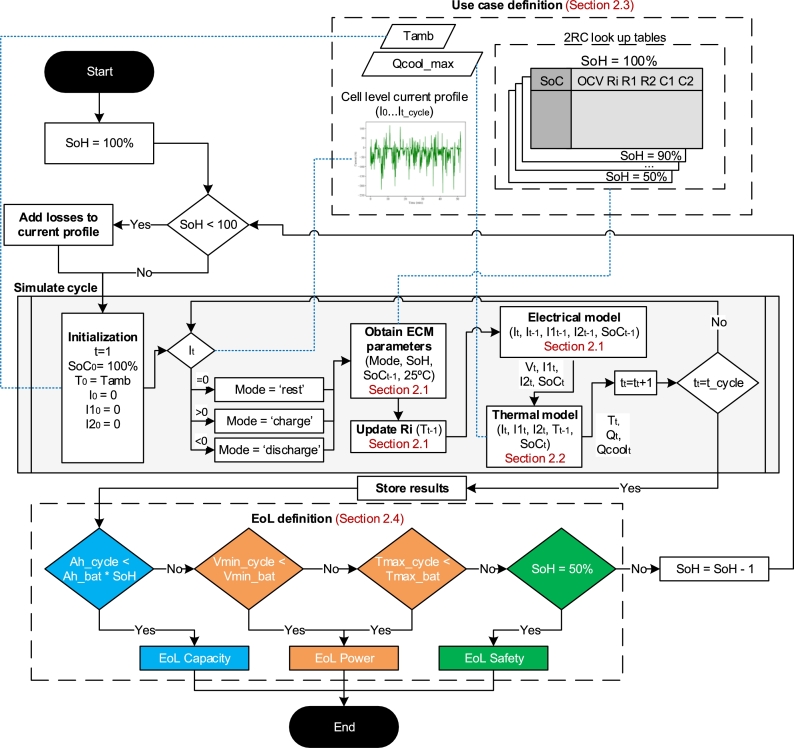
Flow diagram of the simulations, refer to the Sections in red for the detailed calculations.

This study performs a set of simulations of common driving use cases at different levels of degradation to derive the individual EoL. To better understand the process a flow diagram is presented in Fig. 1. The simulations are performed employing degradation aware electrical and thermal models, which are presented in Sections 3.1 and 3.2, respectively. These models allow to obtain the response of the battery for a given use case.

These use case definition provides the inputs for the simulations (the ambient temperature, the cell level current profile and the look-up tables for the electric model) and are detailed in Section 2.3.

Following the flow diagram in Fig. 1, each of the use cases, the simulations are performed starting at 100% SoH until and underperformance is observed, which defines the EoL SoH and the EoL constraint. These EoL constraints are related to capacity, power and safety and are detailed in Section 2.4.

### 2.1 Electrical model

**Figure 2:**
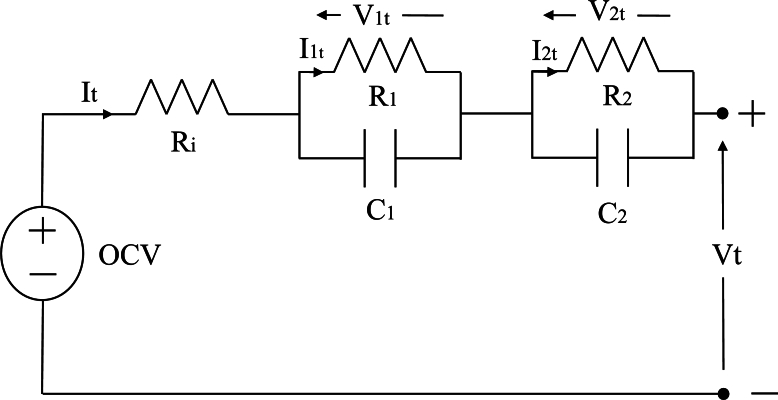
The 2RC ECM employed.

Various electrical models can be used to represent battery behaviour. The most extended model in the BMS for automotive purposes is the Equivalent Circuit Model (ECM) as it provides a simple and efficient way to model battery dynamics [46]. ECMs represent batteries using a combination of ideal circuit elements such as voltage sources, resistors, and capacitors. Other alternatives to the ECM include electrochemical models that, simulate physical phenomena such as the movement of ions and electrons and chemical reactions at the electrodes [47]. However, these models require extensive input data and are computationally intensive. For that reason, in this study, a 2RC ECM is used, combined with a thermal simulation that updates the electrical model parameters and can be used to analyse EoL constraints derived from the temperature. The ECM model used in this study is represented in Fig. 2.

The voltage $V_{t}$ at time *t* can be found solving the electrical circuit, as expressed by Eq. (1).

(1) $$V_{t}=OCV+R_{i}\cdot I_{t}+R_{1}\cdot I_{1,t}+R_{2}\cdot I_{2,t}$$

The current flowing through each of the resistances (*j*=1,2) of the RC branches at time *t* can be expressed by Eq. (2) [48].

(2) $$I_{j,t}=e^{\frac{-\Delta t}{R_{j}\cdot C_{j}}}\cdot I_{j,t-1}+(1-e^{\frac{-\Delta t}{R_{j}\cdot C_{j}}})\cdot I_{t-1}$$

The values of the model parameters (*OCV*, $R_{i}$, $R_{1}$, $R_{2}$, $C_{1}$ and $C_{2}$) are taken from an existing study by Seger et al. [49]. Their work provides the look-up tables of a 2RC model for a 2.1Ah Li-ion cell with a NMC + LMO cathode at 25 °C. Two cycling conditions during the first life were considered, one at 25 °C (FL25 °C) and another at 0 °C (FL0 °C). For the detailed tables of the parameter values, FL25 °C and FL0 °C, the reader can refer to Appendix A and Appendix B of the aforementioned study, respectively [49].

**Figure 3:**
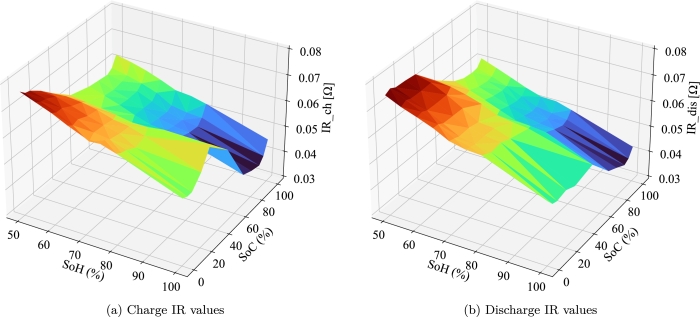
IR values for different SoH and SoC for the FL25 °C case.

The parameter models are provided for different State of Charge (SoC) and SoH values at 25 °C. The SoC values included cover the entire range of [0-100%]. However, since their study aims to evaluate the second-life of the cell, the SoH range considered is limited to [50-80%]. An extrapolation has been made to find the values for 100% SoH considering the tendencies found. This assumption has been carried out knowing that the performance of the first-life batteries above 80% SoH is well known and is not critical. Fig. 3 shows the surface plot for the charge and discharge IR, for the FL25 °C case. Notice that the values for 100% SoH are obtained from a linear regression, which shows a good fit to the data and is in line with the reviewed literature [50]. The R^2^ values of the regressions for the charge and discharge IR range from 0.92 to 0.98 depending on the SoC. Fig. 3 illustrates how the IR, for both charge and discharge, increases linearly with the capacity fade. The rest of the parameters do not show such clear tendencies and therefore, the values corresponding to a SoH 80% have been used for higher SoH levels, as a conservative approach.

As further explained in Section 3.2, a thermal simulation is performed to evaluate the temperature response of the battery. Studies show that the IR is a function of the temperature and that it tends to increase at low temperatures. The relationship of the IR with the temperature follows Eq. (3) and is obtained from the literature [51] where the reference IR ($R_{i,Tref}$) is the one at 25 °C obtained from Seger et al. [49].

(3) $$R_{i_{T}}=R_{i,Tref}\cdot (0.00197\cdot T^{2}-0.1469\cdot T+3.2592)$$

### 2.2 Thermal model

In order to evaluate the temperature of the battery, the heat generation is estimated. The total generated heat in a battery consists of the irreversible heat ($Q_{irr}$) and the reversible heat inside the battery ($Q_{rev}$) [52], as shown by Eq. (4).

(4) $$Q_{t}=Q_{irr,t}-Q_{rev,t}$$

As current flows through the battery, the cell heats up according to Joule's law. The irreversible heat is calculated with Eq. (5) considering the heat dissipated in each of the resistances of the ECM model.

(5) $$Q_{irr,t}=I_{t}^{2}\cdot R_{i}+I_{1,t}^{2}\cdot R_{1}+I_{2,t}^{2}\cdot R_{2}$$

The reversible heat generation is calculated from Eq. (6) where dE/dT is the entropy coefficient (V/K) that depends on the SoC as given by Eq. (7) [52].

(6) $$Q_{rev,t}=I_{t}\cdot (T_{t}\cdot {\left(\frac{dE}{dT}\right)}_{t})$$

(7) $${\left(\frac{dE}{dT}\right)}_{t}=(-0.342+0.979\cdot SoC_{t}-1.49\cdot SoC_{t}^{2}+0.741\cdot SoC_{t}^{3})\cdot 10^{-3}$$

Considering that the battery is equipped with a cooling system that extracts part of the generated heat, the temperature in the battery at time t can be calculated using Eq. (8).

(8) $$T_{t}=T_{t-1}+\frac{(Q_{t}-Q_{cool,t})\cdot dt}{m\cdot C_{p}}$$

where $C_{p}$ is the specific heat capacity of the battery, *m* is the mass, *dt* is the delta of time between the estimated temperature $T_{t}$ and the previous one $T_{t-1}$ and $Q_{cool,t}$ is the cooling power from the thermal management system.

The mass of the cell has been estimated in 55 g for a 18650 cell. The specific heat capacity is obtained from a study conducted to evaluate the heat capacity of a 18650 NCM cell which showed the evolution of $C_{p}$ over the SoC and the temperature [53]. According to the results of the study, the differences due to the SoC can be neglected and the $C_{p}$ shows a linear tendency with the temperature as shown by Eq. (9), where $C_{p}$ is in J/kgK and *T* is in K.

(9) $$C_{p}=1003.6+2.2416\cdot (T-300)$$

The cooling power $Q_{cool,t}$ is calculated to make sure that the battery does not exceed the operating temperature of 35 °C [52]. The nominal power of commercial cooling systems ranges between 5-9 kW [54]. For the simulations, the value of $Q_{cool,t}$ is not allowed to exceed 5 kW for batteries below 40 kWh and 9 kW for the rest.

Even though it is acknowledged that the electrical and thermal model parameters do not pertain to the same type of battery, it is adequate for illustrating the kind of response that the BMS measurements would offer. This aligns with the aim of the study and serves as the basis for the selected approach.

### 2.3 Use cases

In order to analyse representative use cases, the approach presented in a previous work is considered [40], where synthetic driving cycles are generated for two road types and different driving times. The roads considered are Semi Urban (SU) and Semi Highway (SH), each containing a mix of road types but a dominant urban or highway one respectively. Regarding the driving times, two values are considered for each road: one that represents the average driving time between charges and another that covers the driving times of 90% of the population. Table 2 shows the description of road type and driving time for each use case.

**Table 2:** Description of the use cases.

Reference	Road	Population covered	Av. driving time
SU_50_	SU	50%	52 min
SU_90_	SU	90%	126 min
SH_50_	SH	50%	44 min
SH_90_	SH	90%	109 min

**Figure 4:**
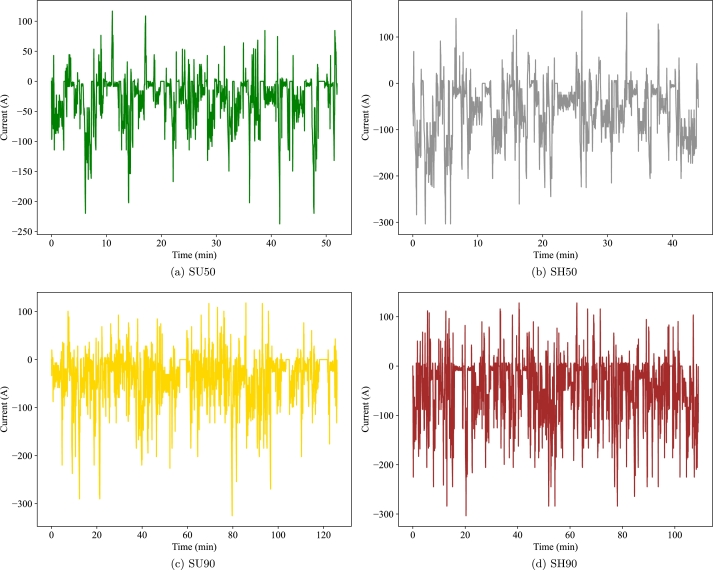
Current profiles of the driving cycles considered.

Fig. 4 shows the current profiles of the four cycles considered where negative currents imply a discharge of the battery and positive ones the regeneration.

To accurately represent existing models on the market, different nominal capacities of the EV battery have been considered. Based on the analysis conducted in a previous study, nominal capacities of 16, 24, 30, 40, 70, and 90 kWh were selected. The useful capacity of the battery is assumed to be 90% of the nominal one. The battery pack configuration is derived considering the cell nominal capacity and voltage and the pack level voltage (320 V) and capacity.

It should be highlighted that in a “real battery” cell-to-cell variations occur, causing imbalances up to 9% [55]. Indeed, the most limiting cell plays a crucial role in defining the overall SoH of the battery pack and the EoL. For this reason, the simulations that are performed at a specific SoH level, refer to the SoH of the most limiting cell. In the entire pack, it could be the case that healthier cells exist, but only the most degraded, showing lowest performance, will limit the battery use.

**Figure 5:**
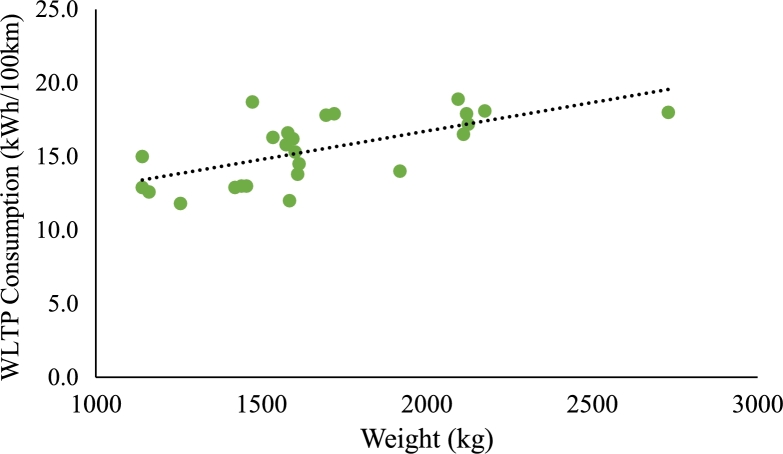
WLTP consumption for different EV weights.

Since different EVs have different specific consumption values, the driving cycles should be modified accordingly. The specific consumption of the EV depends on many factors, like the aerodynamics or efficiencies of the electronic components. However, one of the most relevant factors is the weight of the vehicle. Generally, EVs with larger batteries and longer ranges tend to have higher weights, which can result in higher specific consumption due to reduced efficiency. This is shown in Fig. 5, where the Worldwide Harmonised Light Vehicles Test Procedure (WLTP) consumption of different EV models is compared to their weight. Considering this, a weight correction factor ($f_{weight}$) is included considering that the reference EV, from which the synthetic driving cycle model was created, has a weight of 1614 kg. The current profile of the driving cycle is multiplied by $f_{weight}$ depending on the nominal battery capacity (*C*) in kWh, as shown by Eq. (10).

(10) $$f_{weight}=0.007812\cdot C+0.671933$$

A critical issue for EV batteries is their operation at cold environments. At low temperatures, the range and power of the batteries can be significantly reduced. This is because battery performance is affected by temperature, with colder temperatures increasing the IR [56]. Additionally, at very low temperatures (below 0 °C), the most common degradation mechanisms for Li-ion batteries are slowed down or not dominant, but others such as dendrite growth and lithium plating become relevant [56].

Therefore it is important to maintain the battery temperature in the adequate range, to slow down the degradation and improve the performance. This may require additional heating that can increase the specific energy consumption. Although, in many cases, the self-heating process of the battery may be enough to increase the battery temperature. Besides controlling the temperature of the battery, at cold climates auxiliary heating services are required to maintain the EV temperature, which uses up some of the battery's energy.

To account for these low temperature effects, each of the use cases previously defined is also simulated at a cold temperature. In this case, the discharge currents are increased by a factor of 29% ($f_{climate}$) obtained from comparing the EV specific consumption at average annual temperatures of 18 °C and 8 °C [39]. The electrical model parameters taken into account for the cold temperature scenario correspond to the FL0 °C case where the dominant degradation mechanism is lithium plating, while for the warmer temperature scenario, the FL25 °C parameters are utilized with loss of active material dominating the degradation.

**Figure 6:**
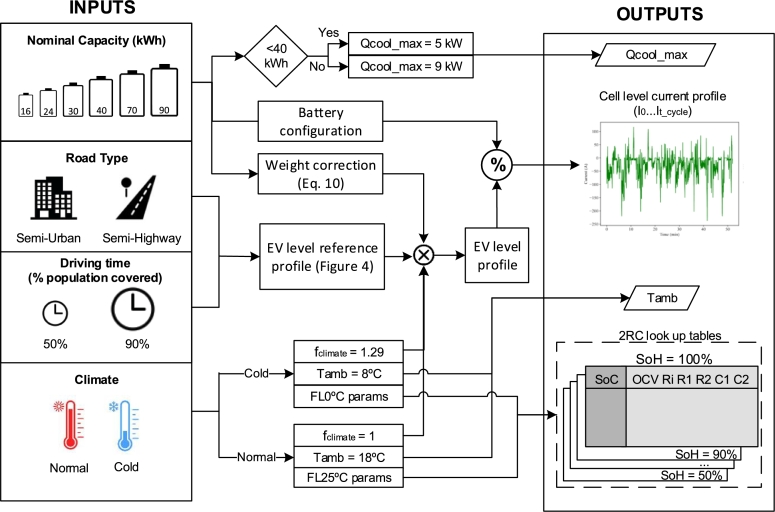
Summary of the use cases and inputs required for the simulations.

To summarize the use cases, a total of 48 simulation use cases are defined considering 6 battery capacities, 4 climates and 4 driver types. The main outputs of the use case definition are summarized in Fig. 6, which are required to run the simulations (see Fig. 1).

Each of the previous use cases is performed at different SoH values [100, ... 50%] or until an EoL constraint is met (as explained in Section 2.4). In order to be able to perform an equivalent driving cycle at lower SoH, the additional heat losses are added to the profile.

### 2.4 End of life requirements

Different issues can translate into an under-performance of the battery and therefore force the EoL. The following constraints have been considered to define the functional EoL for each of the use cases simulated. It should be highlighted that the approach considers cell-to-cell voltage control, since it is the standard for current EV BMS's and that the objective of this study is not to explore the BMS's potential reactions to the constraints, but rather to detect at what point they occur.

• Considering the capacity fade, if at some level of degradation, the available capacity is lower than the required one to cover the driving trip, the battery is considered to reach the EoL for capacity constraints.
• If any cell of the battery pack reaches the minimum operating voltage, the BMS acts to avoid this and therefore limits the power. In this case, the battery is considered to reach the EoL for power constraints.
• If the battery reaches its maximum cooling capability, the temperature will increase above the allowed limit. For that reason the BMS may reduce the available power to avoid the increased temperature. Considering this, if the temperature increases over the limit, the battery is considered to reach the EoL for power constraints.
• If the battery is able to perform the driving cycle without any of the previous constraints at 50% SoH, other safety aspects should be considered, like entering the ageing knee which can create short-circuits [57]. In these cases, the battery is considered to reach the EoL for safety constraints. It should be noted that safety aspects should be considered during the entire operation of the battery and not just at 50% SoH. This could be evaluated with a detailed physical model but not with the ECM approach considered in this study. Nevertheless, the safety issue is well controlled at healthy states and it is only at the ageing knee where the risk for short-circuit and other incidents increases. Studies show that batteries used for EVs show the ageing knee at low SoH values. For example in a recent study, the SoH of automotive cells in the ageing knee varied from 45% to 50% [57]. Therefore it has been assumed that no major safety issues will arise before 50% SoH.

## 3 Results

In this section, the results of the simulations are presented. Since existing literature models have been employed, the novelty of this study is not the detailed analysis of the battery response. Nevertheless, it is necessary to present these results to understand the constraints that force the EoL. First the output voltage of the electrical model is shown in Section 3.1, specifying if any of the use cases reaches the EoL for voltage or capacity constraints. Then the output of the thermal model is shown in 3.2 to evaluate whether the temperature increase may force the battery EoL. Finally, the main focus of the study is presented in 3.3 which shows the EoL SoH values and EoL.

### 3.1 Electric simulations

**Figure 7:**
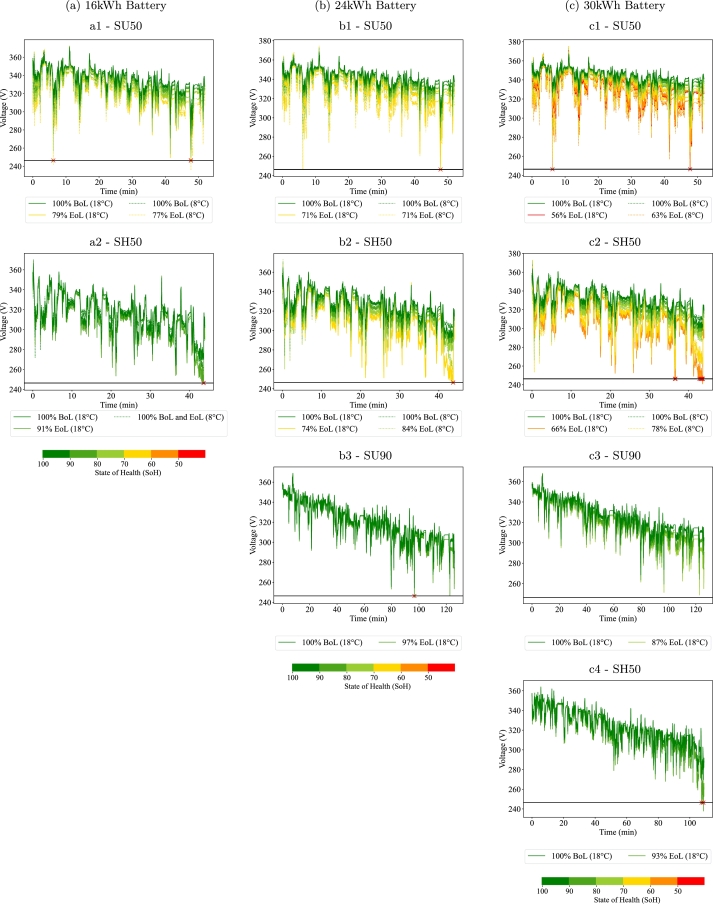
Voltage responses of the small batteries (<40 kWh). *Figures labelled with a, b and c represent the 16, 24 and 30 kWh batteries respectively. The label number 1, 2, 3, 4 represent cycles SU50, SH50, SU90, SH90 respectively. Notice that blank spaces are left if the battery cannot perform the cycle at BoL.*

The voltage responses of the small batteries (below 40 kWh) are shown in Fig. 7. The legend of each plot shows the value of the SoH at the EoL. The dashed lines represent the cold climate simulations and the continuous ones the regular climate simulations. Each of the rows (labelled with number 1, 2 and 3) represents one of the cycles, starting from the SU50 cycle and the columns (labelled with a, b, c) represent the capacity. The voltage responses presented in this section allow to evaluate the power constraints that should be considered when defining the EoL and detect the most demanding conditions for this.

For the SU50 cycle and both climates, all three batteries reach the minimum voltage at some point meaning that they show power constraints before capacity ones. These constraints appear around the minutes 7 and 48 of the trip, when the driver requests a peak of power (see Fig. 4). Notice that at the beginning of the trip, the battery is more charged, but the temperature is lower, especially for the cold climate, as shown in Section 3.2. These low temperatures make the IR larger and therefore the battery is more prone to reaching the minimum voltage. As the battery capacity increases and the current flowing through each cell decreases, the minimum voltage is reached at lower SoH values. The EoL SoH ranges from 79% to 56% for the SU50 case simulated with the small batteries. Therefore, the peak powers requested by the user should be considered to define the most demanding operation, but not necessarily at the end of the trip due to thermal effects.

In the case of the SH50 cycle, some of the simulations are stopped when reaching the minimum voltage and others when the battery capacity is not enough to cover the trip. For this cycle, the power constraints appear towards the end of the trip. In this case, the peak currents are not the largest of the cycle, but the voltage of the battery is low enough to pose problems. Compared with the SU50 cycle, the EoL SoH values are more restrictive and range from 99% to 66%. In fact, the 16 kWh battery is only able to cover the cycle at cold climate at 100% SoH.

For the SU90 cycle, the 16 kWh capacity is too small to cover the trip even at Beginning of Life (BoL). The 24 and 30 kWh batteries are able to perform the cycle at the regular climate and reach the EoL for power or capacity constraints at 97% and 87% SoH, respectively.

Out of the small batteries, only the 30 kWh battery is able to cover the SH90 cycle, but only at the regular climate and only until 93% SoH where power constraints appear, almost at the end of the trip.

**Figure 8:**
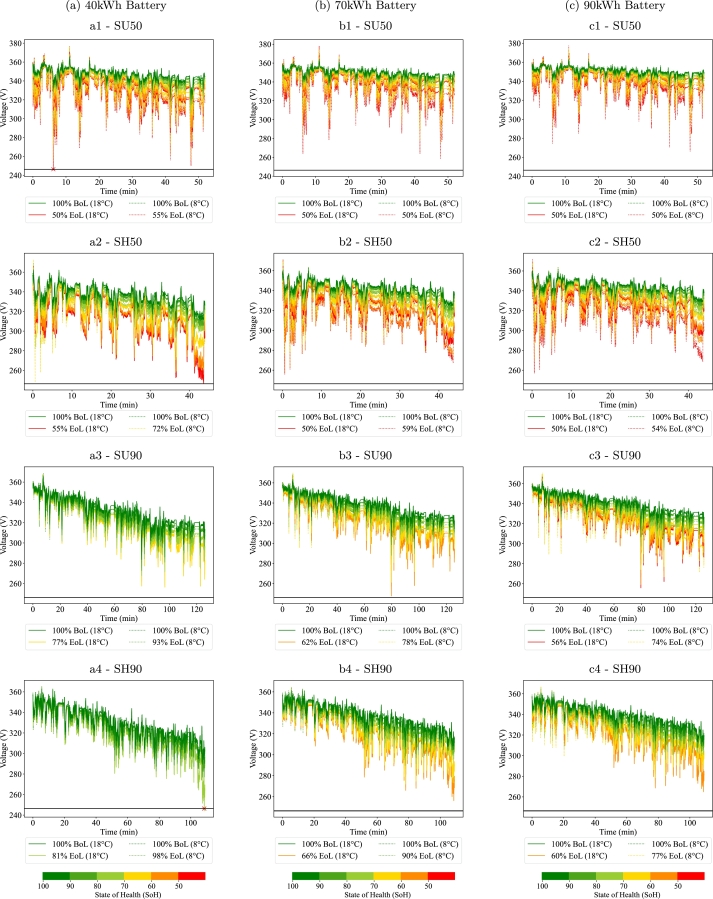
Voltage responses of the large batteries (>=40 kWh). *Figures labelled with a, b and c represent the 40, 70 and 90 kWh batteries respectively. The label number 1, 2, 3, 4 represent cycles SU50, SH50, SU90, SH90 respectively.*

Fig. 8 shows the voltage responses of the large batteries (40 kWh and above). All three of these batteries are able to perform the SU50 cycle even at very degraded stages. In fact, from the simulations it can be derived that their EoL takes place due to safety considerations when reaching 50% SoH and not power constraints as the voltage is always far from the lower threshold. The only exception is the 40 kWh battery at the cold climate where power constraints take place at 55% SoH.

Similarly, for the SH50 cycle, the large batteries can provide the required functionalities until 50-60% SoH except for the 40 kWh at the cold climate, where the EoL takes place at 71% SoH where the battery does not have enough range. For the rest, capacity or safety constraints tend to be the dominant reason to reach the EoL.

For the SU90 and SH90 cycles power constraints do not tend to appear before capacity ones. The only case where this happens is the SU90 cycle performed by the 40 kWh battery at the regular climate at 81% SoH. For the rest of the cases, the EoL SoH ranges between 59% to 92%.

### 3.2 Thermal simulations

**Figure 9:**
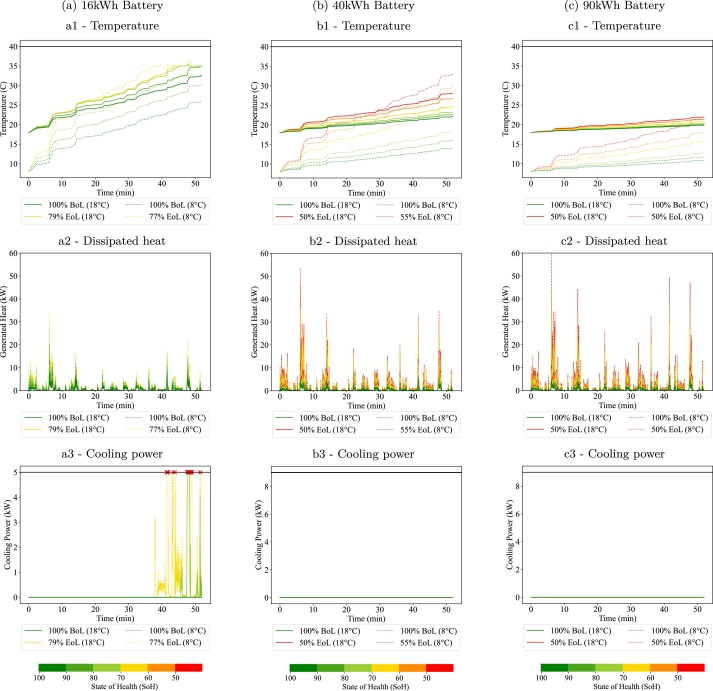
Thermal responses for the SU50 cycle. *Figures labelled with a, b and c represent the 16, 40 and 90 kWh batteries respectively. The label number 1, 2, and 3 represent the temperature, dissipated heat and cooling power respectively. Note that empty figures b3 and c3 imply that no cooling power was required.*

Regarding the thermal response of the batteries, none of the cases simulated reaches the threshold of 40 °C. This means that the considered cooling power is in all cases enough to maintain an adequate operating temperature and that neither power or safety constraints appear due to the temperature increase. As an example, Fig. 9 shows the temperature, generated heat and cooling power for the SU50 cycle and three battery sizes (16, 40 and 90 kWh).

Considering the temperature profiles, it is clear that the temperature increases faster for the smaller batteries. In fact, the 16 kWh battery arrives close to the maximum temperature at the end of the trip, where the cooling system turns on to maintain the temperature at 35 °C. It is noticeable for the simulations at the lowest SoH values, the maximum cooling power is reached and the temperature starts increasing over 35 °C. However this increase is not high enough to force a power limitation by the BMS. This is the case for the simulated cell, however, other chemistries may show a different thermal behaviour, so the over-temperature effect should be considered when analysing the EoL.

For the 40 kWh and 90 kWh batteries, the temperature increase is smaller and the thermal management system does not need to activate the cooling at any point. For higher battery capacities, the current flowing per cell is smaller and therefore the generated heat by the Joule effect is smaller. Notice that, according to the figure, the generated heat increases with the battery capacity. This is because the plots show the total generated heat and, even if the cell level heat generation is smaller, larger-capacity batteries have more cells making the total heat generation higher.

The temperature results of this study are in line with other studies [58]. To understand temperature related constraints, a well known phenomena can be highlighted. The temperature increase is faster when starting the cycle at lower temperatures. At low temperatures two factors increase the temperature jump: the IR is higher and the specific heat capacity decreases. Finally, when comparing the temperature profiles for different degradation levels, it is clear that, as the battery SoH decreases, the battery heats up faster. This is mainly caused by the increased IR and additional heat losses. Therefore, even if the ambient temperature is low, temperature related aspects should not be neglected in the EoL analysis.

### 3.3 EoL constraints

**Figure 10:**
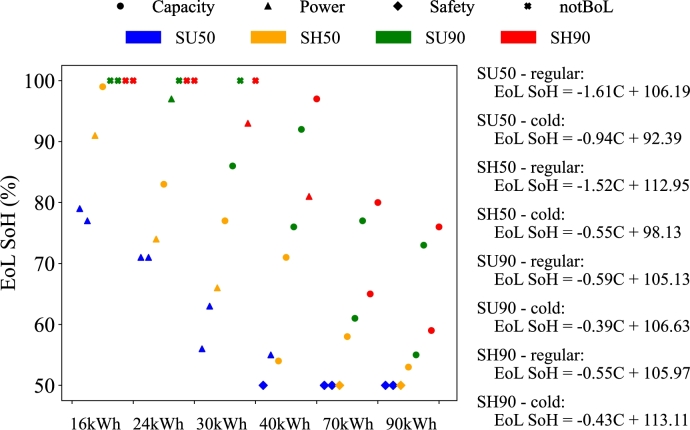
EoL SoH and constraint for all the use cases.

From the simulation results from the previous sections it is possible to obtain a representation of the functional EoL that effectively captures the individualities of each EV. Fig. 10 shows the EoL SoH and constraint for each of the use cases simulated. Notice that the marker shape refers to the EoL constraint and the colour to the cycle. The regressions for each cycle are also included in Fig. 10, to estimate for each cycle and climate, the EoL SoH (%) based on the battery capacity (kWh). Notice how the slope for the SU50 and SH50 cycles for the regular climate are the highest. This shows how, for these use cases, the EoL SoH reaches low values for medium battery capacities.

The linear regressions taken from the simulations prove that, the larger the battery, the softer is the EoL slope depending on the driving cycle. This means that the size of the battery softens the battery exigences as, to perform the same trips, the current through each cell reduces and, as a consequence, so does the self-heating and the cooling needs.

The cases marked with a cross represent the batteries that are not able to perform the trip at the BoL. This happens for the small capacity batteries and the more extreme cycles covering 90% of the population (SU90 and SH90). As the battery capacity increases, the EoL SoH for the same cycle decreases, even arriving to 50% for the largest ones. The most demanding cycle is the SH90 and the least is the SU50.

Another finding can be extracted from Fig. 10, which shows that if the battery presents a power constraint at a low capacity, eventually, this translates into a capacity constraint with a larger capacity, or a safety one if it reaches 50% SoH.

Generally, the EoL is more restrictive for the cold climates. As the regressions also show, the slope for the same cycle decreases for the cold environment compared to the regular one.

Based on the results, it is possible to evaluate the accuracy of the fixed EoL threshold for the performed simulations. Only a quarter of the simulated cases have their functional EoL at 70-80% SoH. When grouping the batteries by capacity, it can be seen that for the small batteries, a quarter of the EoL constraints are found above 80% SoH and a small share have their EoL below 70% SoH. For the larger batteries, the majority of the EoL SoH values are found below 70% and only a small share of the cases are above 80%, which happens for the 40 kWh battery. In addition, the cases where the battery cannot cover the trip in the BoL represent the 18.8% of the total simulated cases. These results are presented in Table 3. Note that, these situations in which the EV cannot cover the trips at BoL are unlikely, as nobody would buy a vehicle that is incapable of meeting the owner's travel needs in the first place.

**Table 3:** EoL SoH share.

Type	100%	Above 80% SoH	70-80%	Below 70%
Small batteries (<40 kWh)	37.5%	25.0%	25.0%	12.5%
Large batteries (>=40 kWh)	0%	12.5%	25.0%	62.5%
**Total**	**18.8%**	**18.8%**	**25.0%**	**37.5%**

Another interesting analysis is related to evaluating the percentage of batteries that reach the EoL for each of the constraints (capacity, power or safety) for the simulated cases. Looking at the last row of Table 4, it can be seen that the leading cause of EoL is a lack of capacity, followed by power constraints, and safety consideration at last. However, the share differs when considering large or small batteries. Small batteries reach the EoL mainly for power constraints. In a few cases capacity limitations are also found. For large batteries the main EoL constraint is the capacity, and power related issues are not common. Almost 30% of the simulated cases for the large batteries reach the EoL with any capacity or power constraints and should be retired for safety considerations.

**Table 4:** EoL constraint share.

Type	Capacity	Power	Safety
Small batteries (<40 kWh)	26.7%	73.3%	0%
Large batteries (>=40 kWh)	62.5%	8.3%	29.2%
**Total**	**48.7%**	**33.3%**	**17.9%**

Notice that the percentages of Table 3 and Table 4 refer to the simulated cases, and not to total amount of retired batteries. These latter percentages depend on different factors. First of all, the evolution of the EV market defines the share of each battery capacity. Considering recent trends, small batteries will hold a small part of the market, while large ones will be dominant. Therefore, the percentages shown in this study corresponding to the large batteries will be imposed over the small ones. Secondly, it should be reminded that the cycles considered in this study represent an average and a more extreme case. The SU90 and SH90 cycles only represent a 10% of the population and therefore, their impact in the overall EoL SoH and constraint share will be less important than the those found for the average cycles (SU50 and SH50).

## 4 Discussion

The results of this study show how the functional EoL for batteries differs from considering a fixed threshold, indicating that, effectively, the fixed EoL threshold approach should be reconsidered. As reviewed in the introduction (Table 1) this generalized assumption affects many studies. For the RUL algorithm case, it is necessary to add another layer to the calculation that takes into account the specific driving requirements of each case. As supported by this study, the particular range and power requirements of each driver severely impact the functional EoL. Regarding second-life assessments and battery stock projections, improved EoL SoH distributions should be included to account for the variability in the retirement conditions.

The presented results show how the functional EoL for the batteries marks the point of degradation where they start presenting performance limitations. However, this does not mean that for a single EV the battery can reach that level of degradation. In fact, it could happen that the EV reaches its EoL first and therefore, the battery is taken out of the vehicle or that the owner sells the EV and the new owner has less demanding requirements. In both cases, this battery could still be functional for a new EV with similar requirements to the first one. A previous study, analysed the expected retirement SoH of different sized batteries, considering that expected EoL of the EV [17], concluding that, as the battery capacity increases, the expected SoH at the EoL is increased, implying that the large capacity batteries reach the EoL at healthier states.

In contrast, this study has shown how, the functional EoL SoH decreases as the battery capacity increases. For example, according to the results of this study, none of the 16 kWh batteries are functional below 60% SoH, which is the expected EoL for most batteries of this capacity according to the aforementioned study. A similar conclusion can be drawn for the 24 kWh battery. To understand these results, it should be highlighted that in this study the same cycles have been adapted to different battery capacities. However, generally, drivers of vehicles with small batteries have less demanding requirements, as they tend to choose the battery based on their needs. In fact, urban cars are bought to drive in the city and rarely travel long distances (not even the SH50 or SU50 profiles considered in this study). Low capacity batteries are most likely going to be selected for requirements below the average ones. Otherwise, the battery would reach the functional EoL before the vehicle, meaning that the driver could notice the underperformance and a battery replacement would be needed.

For the largest capacities (70 and 90 kWh), most batteries are expected to reach the EV EoL over 85% SoH [17]. Considering the results of this study, the batteries at this point of degradation are still able to cover the first-life requirements, and therefore they would be retired from the vehicle while still functional.

Therefore, the small batteries used for relatively demanding applications have the risk of suffering a potential underperformance of the vehicle that forces a replacement and implies additional cost. The large batteries, for most cases, would be extracted from the EV with important residual value and could be reused directly in a different EV, or in a second-life stationary application. However, as mentioned in the introduction and supported by the literature other strategies should be encouraged first before counting on reuse or repurposing, namely Vehicle to Grid [59]. Intermediate capacity batteries allow to avoid battery replacement and can extract all the value of the battery during the first-life, as encouraged by the circular economy.

The findings presented in this study address specific use cases, enabling the discussion of the limitations of the fixed EoL threshold. Nevertheless, real-world scenarios exhibit a broader spectrum of driving conditions and demands. The EoL estimation demonstrated in this study for simulated scenarios can be readily applied to actual EVs by analyzing the data collected by the BMS. In this study, various battery capacities of a particular chemistry are simulated based on literature-derived thermal and electrical models. In practice, each EV model features unique battery configurations, chemistries, and characteristics. For example, the battery chemistry directly defines the performance and degradation rate under a particular usage [60]. Nevertheless, it should be highlighted that the methodology to obtain an accurate EoL is chemistry agnostic and can be achieved by harnessing the comprehensive dataset provided by the BMS.

BMS measurements encompass current, voltage, and temperature, collectively constituting the essential data required for precise EoL predictions. To elaborate further, the battery's current state is captured by the BMS data at any given moment, providing insights into its health in terms of capacity and IR.

Additionally, historical BMS data holds significance in two fundamental ways. On one hand, it contains driving requirements data, encompassing metrics such as energy consumption per trip and peak power demands. As demonstrated in this study, the battery's ability to meet these demands directly influences functionality, ultimately dictating the EoL. The connection between driving requirements and battery health defines the battery's functionality, which can be more precisely quantified by introducing the concept of State of Function (SoF). The SoF diminishes as the battery approaches the EoL and depends on the individual user requirements, unlike the SoH.

On the other hand, historical BMS measurements contain vital information regarding the stress factors that govern the battery's degradation rate. By employing an appropriate degradation model, it is possible to project the degradation trends induced by these stress factors up to EoL. This approach moves beyond traditional RUL algorithms, which oversimplify the task by assuming an EoL fixed threshold at 70-80% SoH. Instead, defining functional and case-specific criteria allows for a more accurate representation of the EoL by defining it at a SoF of 0%.

Following the proposed methodology, once the functional EoL point is estimated, as a point in time or as an amount of mileage reached, it can be compared with the expected lifetime of the vehicle to evaluate whether the battery capacity is the adequate one. Based on this assessment, an individual and data-driven approach can be promoted to maximize the battery usage, decreasing its environmental impact.

## 5 Conclusions

This study challenges the prevailing criteria regarding the EoL for EV batteries that assumes that the fixed threshold of 70-80% SoH adequately captures their lifespan. Through a series of electro-thermal simulations, conducted across various road types, nominal capacities, driving times, and climatic conditions, this study showcases the degradation level at which functional constraints appear.

Only a quarter of the simulated cases actually conform to the fixed threshold, with a significant portion experiencing performance constraints well below the anticipated SoH level. This discrepancy is particularly important for batteries with nominal capacities of 40 kWh and above, which currently dominate the market. Thus, adhering to the fixed threshold generally results in an underestimation of the capabilities of the batteries. These results underscore the necessity of moving beyond the fixed EoL threshold, originally devised for the early EV technology with battery capacities around 24 kWh.

Capacity and power constraints related to undervoltage have shown to force the battery EoL and, in some cases, safety aspects have been considered when the battery reaches 50% SoH. The thermal simulations have shown that the adequate temperature can be maintained even with a high increase in IR. For low-capacity batteries (below 40 kWh) power constraints appear before capacity ones, while for the large batteries, capacity ones are the dominant. This underscores the importance of understanding both the capacity and power required from the driver and estimating the capacity fade and IR increase independently.

Although the simulations have been performed for a specific chemistry, the outcome of the study prevails. The battery EoL should be defined based on the specific application, including both capacity and power requirements, instead of the universal threshold. This study proposes the use of the SoF, as an indicator of battery functionality. The estimation of the SoF requires analysing historical usage data to understand driving requirements, and comparing them to the level of degradation, which can be done employing the data coming from the BMS. This allows to estimate how far the battery is from showing a functional constraint. Future work of this study includes the detailed development and implementation of the SoF estimation employing battery data.

The data-driven algorithms developed to estimate the battery functionality and EoL provide an important tool for sustainable decision-making to promote actions encompassed in the circular economy. As the results of this study highlight, the available data from EVs is an invaluable resource with the potential to drive significant advancements in sustainability, maximize vehicle utilization, and extend battery lifetimes. These actions aim to minimize the use of resources and, in the context of this study, include the selection of the minimum battery capacity that can cover the required functionalities and the maximization of the first-life usage by defining a functional EoL.

## Funding

This project has received funding from the European Union's Horizon 2020 research and innovation program under grant agreement No. 963580. This funding includes funds to support research work and openaccess publications.

## CRediT authorship contribution statement

**Maite Etxandi-Santolaya:** Writing – review & editing, Writing – original draft, Visualization, Validation, Software, Resources, Methodology, Investigation, Formal analysis, Data curation, Conceptualization. **Lluc Canals Casals:** Writing – review & editing, Validation, Supervision, Methodology, Investigation, Funding acquisition, Conceptualization. **Cristina Corchero:** Validation, Supervision, Project administration, Funding acquisition, Conceptualization.

## Declaration of Competing Interest

The authors declare that they have no known competing financial interests or personal relationships that could have appeared to influence the work reported in this paper.
